# Assessment of the Appropriateness of Antimicrobial Use in US Hospitals

**DOI:** 10.1001/jamanetworkopen.2021.2007

**Published:** 2021-03-18

**Authors:** Shelley S. Magill, Erin O’Leary, Susan M. Ray, Marion A. Kainer, Christopher Evans, Wendy M. Bamberg, Helen Johnston, Sarah J. Janelle, Tolulope Oyewumi, Ruth Lynfield, Jean Rainbow, Linn Warnke, Joelle Nadle, Deborah L. Thompson, Shamima Sharmin, Rebecca Pierce, Alexia Y. Zhang, Valerie Ocampo, Meghan Maloney, Samantha Greissman, Lucy E. Wilson, Ghinwa Dumyati, Jonathan R. Edwards, Nora Chea, Melinda M. Neuhauser

**Affiliations:** 1 Division of Healthcare Quality Promotion, Centers for Disease Control and Prevention, Atlanta, Georgia; 2 Lantana Consulting Group, Thetford, Vermont; 3 Department of Medicine, Emory University, Atlanta, Georgia; 4 Georgia Emerging Infections Program, Decatur; 5 Tennessee Department of Health, Nashville; 6 Department of Health Policy, Vanderbilt University School of Medicine, Nashville, Tennessee; 7 Department of Infectious Diseases, Western Health, Melbourne, Victoria, Australia; 8 Colorado Department of Public Health and Environment, Denver; 9 Medical Epidemiology Consulting, Denver, Colorado; 10 Department of Healthcare Management, University of Denver, Colorado; 11 Minnesota Department of Health, St Paul; 12 Hennepin County Public Health, Minneapolis, Minnesota; 13 California Emerging Infections Program, Oakland; 14 New Mexico Department of Health, Santa Fe; 15 Center for Biologics Evaluation and Research, US Food and Drug Administration, Silver Spring, Maryland; 16 Infection Prevention and Control Department, University of New Mexico Hospital, Albuquerque; 17 Oregon Health Authority, Portland; 18 Connecticut Emerging Infections Program, Hartford and New Haven; 19 Department of Medicine, Columbia–New York Presbyterian Hospital; 20 Maryland Department of Health, Baltimore; 21 University of Maryland Baltimore County, Baltimore; 22 New York Emerging Infections Program, Rochester; 23 University of Rochester Medical Center, Rochester, New York

## Abstract

**Importance:**

Hospital antimicrobial consumption data are widely available; however, large-scale assessments of the quality of antimicrobial use in US hospitals are limited.

**Objective:**

To evaluate the appropriateness of antimicrobial use for hospitalized patients treated for community-acquired pneumonia (CAP) or urinary tract infection (UTI) present at admission or for patients who had received fluoroquinolone or intravenous vancomycin treatment.

**Design, Setting, and Participants:**

This cross-sectional study included data from a prevalence survey of hospitalized patients in 10 Emerging Infections Program sites. Random samples of inpatients on hospital survey dates from May 1 to September 30, 2015, were identified. Medical record data were collected for eligible patients with 1 or more of 4 treatment events (CAP, UTI, fluoroquinolone treatment, or vancomycin treatment), which were selected on the basis of common infection types reported and antimicrobials given to patients in the prevalence survey. Data were analyzed from August 1, 2017, to May 31, 2020.

**Exposure:**

Antimicrobial treatment for CAP or UTI or with fluoroquinolones or vancomycin.

**Main Outcomes and Measures:**

The percentage of antimicrobial use that was supported by medical record data (including infection signs and symptoms, microbiology test results, and antimicrobial treatment duration) or for which some aspect of use was unsupported. Unsupported antimicrobial use was defined as (1) use of antimicrobials to which the pathogen was not susceptible, use in the absence of documented infection signs or symptoms, or use without supporting microbiologic data; (2) use of antimicrobials that deviated from recommended guidelines; or (3) use that exceeded the recommended duration.

**Results:**

Of 12 299 patients, 1566 patients (12.7%) in 192 hospitals were included; the median age was 67 years (interquartile range, 53-79 years), and 864 (55.2%) were female. A total of 219 patients (14.0%) were included in the CAP analysis, 452 (28.9%) in the UTI analysis, 550 (35.1%) in the fluoroquinolone analysis, and 403 (25.7%) in the vancomycin analysis; 58 patients (3.7%) were included in both fluoroquinolone and vancomycin analyses. Overall, treatment was unsupported for 876 of 1566 patients (55.9%; 95% CI, 53.5%-58.4%): 110 of 403 (27.3%) who received vancomycin, 256 of 550 (46.6%) who received fluoroquinolones, 347 of 452 (76.8%) with a diagnosis of UTI, and 174 of 219 (79.5%) with a diagnosis of CAP. Among patients with unsupported treatment, common reasons included excessive duration (103 of 174 patients with CAP [59.2%]) and lack of documented infection signs or symptoms (174 of 347 patients with UTI [50.1%]).

**Conclusions and Relevance:**

The findings suggest that standardized assessments of hospital antimicrobial prescribing quality can be used to estimate the appropriateness of antimicrobial use in large groups of hospitals. These assessments, performed over time, may inform evaluations of the effects of antimicrobial stewardship initiatives nationally.

## Key Points

**Question:**

What percentage of hospital antimicrobial use in the US deviates from recommended practices, such as treatment selection or duration, on the basis of medical record documentation?

**Findings:**

In this cross-sectional study of 1566 patients at 192 hospitals, antimicrobial use deviated from recommended practices for 55.9% of patients who received antimicrobials for community-acquired pneumonia or urinary tract infection present at admission or who received fluoroquinolone or intravenous vancomycin treatment.

**Meaning:**

The findings suggest that standardized assessments of hospital antimicrobial prescribing quality can be used to estimate the appropriateness of antimicrobial use in large groups of hospitals.

## Teaser

This cross-sectional study evaluates the appropriateness of antimicrobial use for hospitalized patients treated for community-acquired pneumonia or a urinary tract infection present at admission or for patients who had received fluoroquinolone or intravenous vancomycin treatment.

## Introduction

Optimizing antimicrobial use is critical to slowing the spread of resistant pathogens. In 2014, the US Centers for Disease Control and Prevention (CDC) called for acute care hospitals to implement antimicrobial stewardship programs with the goal of improving antimicrobial use to optimize infection cure rates and minimize harms.^1^ In 2014 and 2015, the White House released the US National Strategy and Action Plan for Combating Antibiotic-Resistant Bacteria, which established antibiotic stewardship outcomes to accomplish by 2020, including a 20% reduction in inappropriate inpatient antibiotic use for monitored conditions and medications.^2,3^ National initiatives have bolstered stewardship efforts in recent years, and data from the CDC’s National Healthcare Safety Network have shown increases in the percentage of hospitals with comprehensive antimicrobial stewardship programs.^1^

Efforts to evaluate antimicrobial stewardship programs’ effect on hospital antimicrobial use typically focus on volume rather than prescribing quality^4,5,6^; it is not clear whether the volume of antimicrobial use correlates with appropriateness.^7^ Prescribing decisions for hospitalized patients are associated with many factors, including comorbidities, allergies, adverse effects, and drug interactions. In addition, the lack of current national treatment guidelines for some infections makes evaluating the appropriateness of US hospital antimicrobial use challenging. Hospital antimicrobial stewards often perform intensive, small-scale medication use evaluations to answer specific questions about appropriateness. Larger-scale evaluations are more difficult to conduct.

We developed and implemented a multicenter objective data collection as part of a hospital prevalence survey of health care–associated infections and antimicrobial use conducted by the CDC’s Emerging Infections Program in 2015. We used these data to assess the appropriateness of antimicrobial use for selected prescribing events in a large group of hospitals and to establish a baseline to which data from subsequent surveys could be compared for estimation of the association of national antimicrobial stewardship interventions with the appropriateness of antimicrobial use at these hospitals.

## Methods

### Hospitals and Patients

This study used data collected by the Emerging Infections Program, which conducted cross-sectional prevalence surveys of health care–associated infections and antimicrobial use in 2011 and 2015 at selected hospitals in 10 states (California, Colorado, Connecticut, Georgia, Maryland, Minnesota, New Mexico, New York, Oregon, and Tennessee); methods and results have been published previously.^8,9,10,11^ Each hospital selected a survey date between May 1 and September 30, 2015. Patients were randomly selected from the census on the morning of the survey date.^8,9,10,11^ The human subjects advisor in the CDC’s National Center for Emerging and Zoonotic Infectious Diseases determined that the survey was a nonresearch public health activity. Emerging Infections Program sites and hospitals determined that the survey was a nonresearch activity or approved it with an informed consent waiver. This study followed the Strengthening the Reporting of Observational Studies in Epidemiology (STROBE) reporting guideline.

### Data Collection

Data collected in the 2011 survey identified 4 common antimicrobial prescribing events for assessment in the 2015 survey: 2 infection-based events, including treatment of community-acquired pneumonia (CAP) or treatment of urinary tract infection (UTI) present at admission, and 2 antimicrobial-based events, including treatment with fluoroquinolones (FQ) or treatment with intravenous vancomycin (VANC). Medical record abstraction forms were developed for each event to compose the antimicrobial quality assessment (AQUA) component of the 2015 survey. Emerging Infections Program staff, who were not required to be clinicians or antimicrobial stewards, reviewed medical records retrospectively to collect information on comorbidities, health care exposures, antimicrobial allergies, illness severity, infections during the hospitalization, microbiology and other test results, and treatment.

Patients were eligible for multiple AQUA data collections based on the antimicrobials given to the patient on the survey date or the previous day and the reported rationale for use. Patients were eligible for CAP data collection if they were 1 year or older, did not have certain underlying conditions or exposures, and were receiving antimicrobials for treatment of CAP on the survey date or the previous day. Patients were excluded from CAP data collection for the following reasons: (1) a stay in a nursing home, long-term care facility, or long-term acute care hospital before admission to the survey hospital; (2) hospitalization for 2 or more days in the 90 days before admission (other than the current admission); (3) receipt of intravenous antimicrobials, cancer chemotherapy, or wound care in the 30 days before admission; (4) requirement for long-term hemodialysis or mechanical ventilation at home; (5) diagnosis of cystic fibrosis or AIDS or another acquired or congenital immunodeficiency; (6) history of solid organ or hematopoietic stem cell transplant; and (7) treatment with high-dose corticosteroids or other immunosuppressive agents for more than 30 days. Patients were eligible for UTI data collection if they were 1 year or older and receiving antimicrobials on the survey date or the previous day for treatment of UTI present at the time of admission to the survey hospital. Patients were eligible for FQ data collection if they were 18 years or older and receiving FQ treatment on the survey date or the previous day, and patients were eligible for VANC data collection if they were 1 year or older and receiving VANC treatment on the survey date or the previous day.

### Statistical Analysis

Data were analyzed from August 1, 2017, to May 31, 2020. Final data downloaded from the prevalence survey data system on November 16, 2017, were analyzed using SAS, version 9.4 (SAS Institute Inc) or OpenEpi, version 3.01.^12^ AQUA analysis pathways were developed by CDC staff^13,14,15^ and refined from 2018 to 2020 with input from an antimicrobial stewardship expert group convened by The Pew Charitable Trusts.

Among patients eligible for AQUA data collection, a subset was eligible for analysis in which the finalized pathways were used. For the CAP pathway, the analysis included patients who (1) were 18 years or older, (2) had radiographic evidence of pneumonia during the first 5 hospital days, (3) had signs or symptoms of pneumonia during the first 2 hospital days, (4) received inpatient pneumonia treatment for 3 or more calendar days, and (5) did not have other infections reported during their hospitalizations. For the UTI pathway, analysis included patients who (1) were not pregnant and did not have neutropenia or a history of transplant, (2) received inpatient UTI treatment for 1 or more calendar days, and (3) did not have other infections reported. For the FQ pathway, analysis included patients with only 1 infection type who received FQ for 1 or more days. For the VANC pathway, analysis included patients with only 1 infection type who received VANC for more than 3 days (eMethods and eFigures 1-4 in the Supplement). We excluded patients from the VANC or FQ pathway if they were included in the CAP or UTI pathway.

Analysis pathways categorized the quality of antimicrobial use for each patient’s AQUA event(s) (eMethods in the Supplement). Because categorizations were based on data collected using standardized forms rather than clinical judgment at the time of medical record review, we used the terms *supported* and *unsupported* as proxies for appropriate and inappropriate or unnecessary use. Antimicrobial use was supported if there was medical record evidence that (1) treatment was clinically indicated for the infection for which the patient had a reported diagnosis, (2) antimicrobial selection was consistent with available guidelines or microbiology data, and (3) duration was consistent with recommendations in available guidelines.^7,16,17,18,19,20^ In cases involving more severe or complicated infections (eg, sterile site infections, sepsis, or infections due to selected pathogens such as mycobacteria), duration was not considered in the determination of whether prescribing was supported. Antimicrobial use for which some aspect was unsupported by medical record data (hereafter referred to as unsupported use) was defined as (1) use of antimicrobials to which the pathogen was not susceptible, use in the absence of signs or symptoms of infection, or use without supporting microbiologic data; (2) use of antimicrobials that deviated from guidelines; or (3) use that exceeded recommended duration.

Percentages of supported vs unsupported use and 95% CIs were calculated for each analysis pathway and across all pathways. For patients included in both the FQ and the VANC analysis pathways, discordant determinations were resolved to a single determination by 1 of the authors (S.S.M.).

## Results

### Patient Eligibility for AQUA Analysis Pathways

Of 12 299 patients, 1566 patients (12.7%) in 192 hospitals were included in AQUA analyses; the median age was 67 years (interquartile range, 53-79 years), and 864 (55.2%) were female. Characteristics of patients included in each analysis pathway are shown in Table 1. Of 12 299 patients included in the survey, 6084 (49.5%) received antimicrobial medications on the survey date or day before, and 4476 of these patients (73.6%) received antimicrobial treatment for infection, as reported previously.^11^ Among 4476 patients receiving antimicrobial treatment for infection, 2680 (59.9%) were eligible for 1 or more AQUA data collections: 430 (9.6% of all patients receiving antimicrobial treatment for infection) in the CAP analysis pathway, 846 (18.9%) in the UTI pathway, 1112 (24.8%) in the VANC pathway, and 1068 (23.9%) in the FQ pathway (eFigures 1-4 in the Supplement). Of the 2680 patients eligible for AQUA data collection, 1566 were included in the analyses: 219 (14.0%) in the CAP pathway, 452 (28.9%) in the UTI pathway, 550 (35.1%) in the FQ pathway, and 403 (25.7%) in the VANC pathway.

**Table 1.** Characteristics of Patients Included in the AQUA Pathways

Characteristic	Patients, No. (%)			
	CAP pathway (n = 219)	UTI pathway (n = 452)	FQ pathway (n = 550)	VANC pathway (n = 403)
Sex				
Female	110 (50.2)	311 (68.8)	295 (53.6)	178 (44.2)
Male	109 (49.8)	141 (31.2)	255 (46.4)	225 (55.8)
Age category, y				
1-17^a^	NA^b^	9 (2.0)	NA^c^	10 (2.5)
18-24	5 (2.3)	12 (2.7)	13 (2.4)	5 (1.2)
25-44	13 (5.9)	40 (8.9)	64 (11.6)	75 (18.6)
45-64	78 (35.6)	76 (16.8)	176 (32.0)	148 (36.7)
65-84	93 (42.5)	216 (47.8)	220 (40.0)	135 (33.5)
≥85	30 (13.7)	99 (21.9)	77 (14.0)	30 (7.4)
Location from which patient was admitted				
Private residence	212 (96.8)	377 (83.4)	476 (86.6)	320 (79.4)
Long-term care facility	NA^c^	64 (14.2)	45 (8.2)	51 (12.7)
Long-term acute care hospital	NA^c^	1 (0.2)	2 (0.4)	3 (0.7)
Another acute care hospital	3 (1.4)	7 (1.6)	21 (3.8)	16 (4.0)
Other	3 (1.4)	0	4 (0.7)	10 (2.5)
Unknown	1 (0.5)	3 (0.7)	2 (0.4)	3 (0.7)
Health care exposures in 30 d before admission				
Intravenous antimicrobials	NA^c^	47 (10.4)	73 (13.3)	93 (23.1)
Cancer chemotherapy	NA^c^	9 (2.0)	33 (6.0)	26 (6.5)
Wound care	NA^c^	15 (3.3)	18 (3.3)	41 (10.2)
Long-term hemodialysis	NA^c^	4 (0.9)	13 (2.4)	11 (2.7)
Surgery	2 (0.9)	22 (4.9)	21 (3.8)	38 (9.4)
None	191 (87.2)	301 (66.6)	334 (60.7)	181 (44.9)
Unknown	26 (11.9)	76 (16.8)	80 (14.6)	58 (14.4)
Hospitalized in the 90 d before admission				
Yes	NA^c^	109 (24.1)	167 (30.4)	164 (40.7)
No	190 (86.8)	277 (61.3)	317 (57.6)	188 (46.7)
Unknown	29 (13.2)	66 (14.6)	66 (12.0)	51 (12.7)
Allergy to antimicrobials reported	58 (26.5)	151 (33.4)	215 (39.1)	115 (28.5)
Any penicillin	35 (16.0)	77 (17.0)	139 (25.3)	71 (17.6)
Severe penicillin^d^	8 (3.7)	18 (4.0)	46 (8.4)	20 (5.0)
Underlying conditions				
Asthma	19 (8.7)	23 (5.1)	46 (8.4)	30 (7.4)
Chronic obstructive pulmonary disease or emphysema	89 (40.6)	56 (12.4)	165 (30.0)	77 (19.1)
Chronic kidney disease	22 (10.1)	89 (19.7)	83 (15.1)	48 (11.9)
Chronic liver disease	4 (1.8)	9 (2.0)	24 (4.4)	19 (4.7)
Congestive heart failure	42 (19.2)	58 (12.8)	85 (15.5)	52 (12.9)
Diabetes	60 (27.4)	159 (35.2)	139 (25.3)	141 (35.0)
HIV infection	3 (1.4)	8 (1.8)	8 (1.5)	6 (1.5)
Malignant neoplasm	20 (9.1)	54 (12.0)	93 (16.9)	67 (16.6)
Other immunosuppression^e^	NA^c^	6 (1.3)	33 (6.0)	29 (7.2)
Urinary tract condition^f^	10 (4.6)	111 (24.6)	26 (4.7)	12 (3.0)
None	28 (12.8)	57 (12.6)	78 (14.2)	58 (14.4)
Unknown	4 (1.8)	0	4 (0.7)	4 (1.0)
Severity of illness				
In intensive care unit during hospitalization	82 (37.4)	64 (14.2)	111 (20.2)	108 (26.8)
Systemic inflammatory response syndrome present^g^	62 (28.3)	85 (18.8)	63 (11.5)	87 (21.6)
Hospital size category				
Small: <150 beds	93 (42.5)	193 (42.7)	223 (40.6)	121 (30.0)
Medium: 150-399 beds	103 (47.0)	205 (45.4)	238 (43.3)	174 (43.2)
Large: ≥400 beds	23 (10.5)	54 (12.0)	89 (16.2)	108 (26.8)
Location of patient in hospital on the survey date				
Critical care unit	49 (22.4)	43 (9.5)	53 (9.6)	55 (13.7)
Mixed acuity unit	5 (2.3)	7 (1.6)	5 (0.9)	3 (0.7)
Specialty care area	0	0	4 (0.7)	2 (0.5)
Step-down unit	10 (4.6)	15 (3.3)	27 (4.9)	25 (6.2)
Ward	155 (70.8)	387 (85.6)	461 (83.8)	318 (78.9)
Central line in place on survey date				
Yes	36 (16.4)	43 (9.5)	91 (16.6)	124 (30.8)
No	183 (83.6)	408 (90.3)	457 (83.1)	279 (69.2)
Unknown	0	1 (0.2)	2 (0.4)	0
Urinary catheter in place on survey date				
Yes	52 (23.7)	138 (30.5)	91 (16.6)	87 (21.6)
No	167 (76.3)	313 (69.3)	457 (83.1)	314 (77.9)
Unknown	0	1 (0.2)	2 (0.4)	2 (0.5)
Ventilator in place on survey date				
Yes	19 (8.7)	10 (2.2)	18 (3.3)	29 (7.2)
No	200 (91.3)	442 (97.8)	530 (96.4)	374 (92.8)
Unknown	0	0	2 (0.4)	0
Antimicrobials given before hospitalization for the current infection				
Yes	32 (14.6)	93 (20.6)	NC	NC
No	172 (78.5)	324 (71.7)	NC	NC
Unknown	15 (6.8)	35 (7.7)	NC	NC
Antimicrobials prescribed at hospital discharge^h^				
Yes	132 (60.3)	257 (56.9)	258 (46.9)	47 (11.7)
No	81 (37.0)	189 (41.8)	286 (52.0)	352 (87.3)
Unknown	6 (2.7)	6 (1.3)	6 (1.1)	4 (1.0)
Duration of antimicrobial treatment, median (IQR), d^i^	10 (8-13)	8 (5-11)	7 (4-11)	7 (5-11)
Hospital length of stay, median (IQR), d	6 (4-11)	4 (3-7)	6 (3-9)	9 (5-15)
Outcome of hospitalization				
Died	11 (5.0)	11 (2.4)	16 (2.9)	18 (4.5)
Survived	208 (95.0)	441 (97.6)	533 (96.9)	384 (95.3)
Unknown	0	0	1 (0.2)	1 (0.3)

Abbreviations: AQUA, antimicrobial quality assessment; CAP, community-acquired pneumonia; FQ, fluoroquinolone; IQR, interquartile range; NA, not applicable; NC, not collected; UTI, urinary tract infection present on admission; VANC, intravenous vancomycin.

^a^ Patients younger than 1 year were not eligible for any AQUA data collection.

^b^ Patients younger than 18 years were not included in the CAP analysis.

^c^ Patients with these characteristics were not eligible for the AQUA event data collection.

^d^ Reactions categorized as severe included anaphylaxis, wheezing, throat tightness, trouble breathing, angioedema, swelling, hives, urticaria, blisters, Stevens-Johnson syndrome, syncope, shock, thrombocytopenia, and liver failure.

^e^ Includes asplenia, long-term corticosteroid or other immunosuppressive therapy, neutropenia, solid organ transplant, or hematopoietic stem cell transplant.

^f^ Includes congenital urinary tract abnormalities, nephrolithiasis, recurrent urinary tract infection, vesicoureteral reflux, ureteral stent, urostomy, and other unspecified urinary tract abnormalities.

^g^ During the first 24 hours of treatment during the hospitalization.

^h^ Includes antimicrobials given to continue treatment of CAP or UTI or treatment with FQ or VANC continued after discharge.

^i^ Total duration of treatment included inpatient treatment plus anticipated postdischarge treatment for CAP or UTI or with FQ or VANC. Data shown reflect only patients for whom inpatient and postdischarge treatment duration were known. Postdischarge treatment duration was available for a subset of patients who were reported to have been prescribed antimicrobials at discharge: for CAP, 114 of 132 patients; for UTI, 207 of 257 patients; for FQ, 206 of 258 patients; and for VANC, 32 of 47 patients.

### Antimicrobial Prescribing Quality Assessment

Among 219 patients in the CAP pathway (Table 2 and eFigure 5 in the Supplement), antimicrobial prescribing was categorized as supported for 45 (20.5%; 95% CI, 15.6%-26.3%) and unsupported for 174 (79.5%; 95% CI, 73.7%-84.4%). Most patients with unsupported CAP treatment were treated for 8 or more days (103 of 174 [59.2%]) or received antimicrobials on inpatient treatment day 3 (68 of 174 [39.1%]) that were inconsistent with current guidelines.

**Table 2.** Percentage of Antimicrobial Treatment Supported or Unsupported Based on Medical Record Documentation in the CAP Analysis Pathway

Pathway criterion	Patients, No. (%) (n = 219)	Prescribing quality determination
**No pathogens identified from respiratory or sterile site cultures in first 5 hospital d**		
All	171 (78.1)	NA
Did not receive guideline-similar CAP treatment on day 3 of inpatient treatment	68 (31.1)	Unsupported
Received guideline-similar CAP treatment on day 3 of inpatient treatment		
All	103 (47.0)	NA
Treatment duration <8 d^a^	32 (14.6)	Supported
Treatment duration ≥8 d^a^	71 (32.4)	Unsupported
**Pathogens identified from respiratory or sterile site cultures in first 5 hospital d**		
All	48 (21.9)	NA
Pathogen not susceptible to antimicrobial treatment^b^	3 (1.4)	Unsupported
Pathogen susceptible to antimicrobial treatment^b^	45 (20.5)	NA
Pathogen cultured from blood, cerebrospinal fluid, or pleural fluid sample	4 (1.8)	Supported
Pathogen not cultured from blood, cerebrospinal fluid, or pleural fluid sample	41 (18.7)	NA
Special pathogen isolated^c^	2 (0.9)	Supported
No special pathogens isolated	39 (17.8)	NA
Treatment duration <8 d^a^	7 (3.2)	Supported
Treatment duration ≥8 d^a^	32 (14.6)	Unsupported
**Total supported and unsupported CAP treatment**		
Supported CAP treatment	45 (20.5)	NA
Unsupported CAP treatment	174 (79.5)	NA

Abbreviations: CAP, community-acquired pneumonia; NA, not applicable.

^a^ Treatment duration was defined as the duration of inpatient treatment plus the anticipated duration of postdischarge treatment. If postdischarge treatment duration was missing, it was estimated by determining the median days of postdischarge treatment among patients in the same step of the analysis pathway with the same duration of inpatient treatment.

^b^ Pathogens were assessed to determine whether they were susceptible (or likely susceptible if no susceptibility data were reported) to at least 1 antimicrobial that the patient was receiving on the day after the microbiology test result was reported to be final.

^c^ Special pathogens were defined as *Mycobacterium* species (other than *Mycobacterium gordonae*), *Aspergillus* species, *Nocardia* species, or other uncommon organisms requiring specialized, prolonged treatment.

Among 452 patients in the UTI pathway (Table 3 and eFigure 6 in the Supplement), antimicrobial treatment was categorized as supported for 105 (23.2%; 95% CI, 19.6%-27.3%) and unsupported for 347 (76.8%; 95% CI, 72.7%-80.5%). Unsupported antimicrobial use was most commonly attributed to lack of documented signs or symptoms of UTI (174 of 347 [50.1%]), continued treatment without qualifying microbiologic evidence of infection (95 of 347 [27.4%]), or excessive treatment duration (74 of 347 [21.3%]).

**Table 3.** Percentage of Antimicrobial Treatment Supported or Unsupported Based on Medical Record Documentation in the UTI Analysis Pathway

Pathway criterion	Patients, No. (%) (n = 452)	Prescribing quality determination
**No signs or symptoms of UTI documented in first 2 hospital d and no matching pathogen isolated from eligible urine and blood cultures**		
All	174 (38.5)	Unsupported
**Signs or symptoms of UTI documented in first 2 hospital d or a matching pathogen isolated from eligible urine and blood cultures**		
All	278 (61.5)	NA
Eligible positive urine or blood culture collected in the first 5 hospital d	171 (37.8)	NA
Pathogen not susceptible to antimicrobial treatment^a^	4 (0.9)	Unsupported
Pathogen susceptible to antimicrobial treatment^a^	167 (36.9)	NA
Only fluoroquinolone treatment given	18 (4.0)	NA
Treatment duration <8 d^b^	4 (0.9)	Supported
Treatment duration ≥8 d^b^	14 (3.1)	Unsupported
Antimicrobials other than fluoroquinolones given	149 (33.0)	NA
Fever documented or eligible positive blood culture result	86 (19.0)	NA
Treatment duration <15 d^b^	66 (14.6)	Supported
Treatment duration ≥15 d^b^	20 (4.4)	Unsupported
No fever documented and no eligible positive blood cultures	63 (13.9)	NA
Treatment duration <8 d^b^	23 (5.1)	Supported
Treatment duration ≥8 d^b^	40 (8.8)	Unsupported
No eligible positive urine or blood culture results in the first 5 hospital d	107 (23.7)	NA
Treatment stopped within 3 d	12 (2.7)	Supported
Treatment continued for >3 d	95 (21.0)	Unsupported
**Total supported and unsupported UTI treatment**		
Supported UTI treatment	105 (23.2)	NA
Unsupported UTI treatment	347 (76.8)	NA

Abbreviations: NA, not applicable; UTI, urinary tract infection.

^a^ Pathogens were assessed to determine whether they were susceptible (or likely susceptible if no susceptibility data were reported) to at least 1 antimicrobial the patient was receiving the day after the microbiology test result was reported to be final.

^b^ Treatment duration was defined as the duration of inpatient treatment plus the anticipated duration of postdischarge treatment. If postdischarge treatment duration was missing, it was estimated by determining the median days of postdischarge treatment among patients in the same step of the analysis pathway with the same duration of inpatient treatment.

Among 550 patients in the FQ pathway (Table 4 and eFigure 7 in the Supplement), antimicrobial prescribing was categorized as supported for 294 (53.5%; 95% CI, 49.3%-57.6%) and unsupported for 256 (46.5%; 95% CI, 42.4%-50.7%), most commonly because of FQ treatment for 8 or more days in patients with lower respiratory tract, abdominal, or gastrointestinal infections without supporting microbiologic data (161 of 256 [62.9%]).

**Table 4.** Percentage of Antimicrobial Treatment Supported or Unsupported Based on Medical Record Documentation in the FQ Analysis Pathway

Pathway criterion	Patients, No. (%) (n = 550)	Prescribing quality determination
**No pathogen identified from a specimen type consistent with the reported infection site**^**a**^		
All	432 (78.5)	NA
Sepsis^b^	49 (8.9)	Supported
Bone or joint infection	3 (0.5)	NA
Signs or symptoms consistent with the reported infection site	3 (0.5)	Supported
No signs or symptoms consistent with the reported infection site	0	Unsupported
Pneumonia; lower respiratory tract infection; or gastrointestinal, hepatobiliary, or intra-abdominal infection without sepsis	320 (58.2)	NA
Signs or symptoms consistent with the reported infection site	314 (57.1)	NA
FQ treatment duration <8 d^c^	153 (27.8)	Supported
FQ treatment duration ≥8 d^c^	161 (29.3)	Unsupported
No signs or symptoms consistent with the reported infection site	6 (1.1)	Unsupported
Other infection site	60 (10.9)	NA
FQ stopped within 3 d if no microbiology testing done or within 1 d of final negative culture or CIDT result^d^	24 (4.4)	Supported
FQ continued	36 (6.5)	Unsupported
**Pathogen identified from specimen type consistent with the site of infection**^**e**^		
All	118 (21.5)	NA
Pathogen not susceptible or likely not susceptible to the FQ received	22 (4.0)	NA
FQ stopped within 1 d of the final culture or CIDT result^d^	16 (2.9)	Supported
FQ continued	6 (1.1)	Unsupported
Pathogen susceptible or likely susceptible to the FQ received	96 (17.5)	NA
Pathogen identified from blood or other sterile site	14 (2.5)	Supported
Pathogen identified from nonsterile site	82 (14.9)	NA
Special pathogen isolated^f^	1 (0.2)	Supported
No special pathogens isolated^f^	81 (14.7)	NA
Signs or symptoms consistent with reported infection site	67 (12.2)	NA
UTI with fever	2 (0.4)	NA
FQ treatment duration <15 d^c^	2 (0.4)	Supported
FQ treatment duration ≥15 d^c^	0	Unsupported
Other infection type	65 (11.8)	NA
FQ treatment <8 d^c^	32 (5.8)	Supported
FQ treatment duration ≥8 d^c^	33 (6.0)	Unsupported
No signs or symptoms consistent with reported infection site	14 (2.5)	Unsupported
**Total supported and unsupported FQ treatment**		
Supported FQ treatment	294 (53.5)	NA
Unsupported FQ treatment	256 (46.5)	NA

Abbreviations: CIDT, culture-independent diagnostic test; FQ, fluoroquinolone; NA, not applicable; UTI, urinary tract infection.

^a^ Includes patients for whom cultures and CIDTs were not performed, patients for whom all culture and CIDT results were negative, and patients for whom culture and CIDT results were positive only for nonpathogens at the site of infection (eg, normal or mixed flora, yeast, or *Candida* species from a urine culture or respiratory tract culture).

^b^ Sepsis was defined using systemic inflammatory response syndrome criteria on the first day of FQ treatment based on 2 or more of the following: (1) temperature lower than 36 °C or higher than 38 °C, (2) heart rate greater than 90 beats per minute, (3) respiratory rate greater than 20 breaths per minute (or partial pressure of carbon dioxide, arterial <32 mm Hg), or (4) white blood cell count less than 4000 cells/mm^3^ or greater than 10 000 cells/mm^3^ or greater than 10% bands in addition to (1) systolic blood pressure lower than 90 mm Hg, mean arterial pressure lower than 65 mm Hg, or receipt of vasopressors or (2) lactate level greater than 2 mmol/L (to convert to milligrams per deciliter, divide by 0.111).

^c^ Treatment duration was defined as the duration of inpatient FQ treatment plus the anticipated duration of postdischarge FQ treatment. If postdischarge treatment duration was missing, it was estimated by determining the median days of postdischarge treatment among patients in the same step of the analysis pathway with the same duration of inpatient treatment. Data on other non-FQ antimicrobials were not collected.

^d^ The time from collection to the final negative culture and CIDT results was estimated using the median time from collection to the final positive culture result.

^e^ Includes results of cultures or CIDTs performed 5 days before the initiation of FQ treatment through the last date of FQ treatment.

^f^ *Mycobacterium* species (other than *Mycobacterium gordonae*).

Among 403 patients in the VANC pathway (Table 5 and eFigure 8 in the Supplement), antimicrobial use was categorized as supported for 293 (72.7%; 95% CI, 68.2%-76.9%) and unsupported for 110 (27.3%; 95% CI, 23.1%-31.8%). Unsupported treatment was commonly attributed to continuation of VANC in patients who did not appear to require it (56 of 110 [50.9%]), for example, patients without susceptible or likely susceptible pathogens identified from microbiologic testing or patients with cultures positive for pathogens susceptible to penicillin, ampicillin, or oxacillin and without a severe or unspecified penicillin allergy.

**Table 5.** Percentage of Antimicrobial Treatment Supported or Unsupported Based on Medical Record Documentation in the Vancomycin Analysis Pathway

Pathway criterion	Patients, No. (%) (n = 403)	Prescribing quality determination
Neutropenia	7 (1.7)	Supported
Cystic fibrosis with a history of MRSA colonization or infection	2 (0.5)	Supported
**No pathogen identified from a specimen type consistent with the reported infection site**^**a**^		
All	214 (53.1)	NA
Sepsis^b^	49 (12.2)	Supported
Bone or joint, cardiovascular, or central nervous system infection without sepsis	16 (4.0)	NA
Signs or symptoms consistent with the reported infection site	16 (4.0)	Supported
No signs or symptoms consistent with the reported infection site	0	Unsupported
Purulent skin and soft-tissue infection without sepsis	19 (4.7)	NA
Vancomycin treatment duration <11 d^c^	15 (3.7)	Supported
Vancomycin treatment duration ≥11 d^c^	4 (1.0)	Unsupported
Health care–associated pneumonia or lower respiratory tract infection without sepsis	55 (13.6)	NA
Signs or symptoms consistent with the reported infection site	55 (13.6)	NA
Vancomycin treatment duration <8 d^c^	36 (8.9)	Supported
Vancomycin treatment duration ≥8 d^c^	19 (4.7)	Unsupported
No signs or symptoms consistent with the reported infection site	0	Unsupported
Other infection site	75 (18.6)	NA
Vancomycin stopped within 3 d if no microbiology testing done or within 1 d of the final negative culture or CIDT result^d^	45 (11.2)	Supported
Vancomycin continued	30 (7.4)	Unsupported
**Pathogen identified from specimen type consistent with the site of infection**^**e**^		
All	180 (44.7)	NA
Pathogen not susceptible or likely not susceptible to vancomycin	31 (7.7)	NA
Vancomycin stopped within 1 d of the final culture or CIDT result^d^	17 (4.2)	Supported
Vancomycin continued	14 (3.5)	Unsupported
Pathogen susceptible or likely susceptible to vancomycin	149 (37.0)	NA
Pathogen identified from blood or other sterile site	37 (9.2)	NA
Pathogen susceptible or likely susceptible to penicillin, ampicillin, or oxacillin	13 (3.2)	NA
Severe or unspecified penicillin allergy	1 (0.2)	Supported
No severe or unspecified penicillin allergy	12 (3.0)	NA
Vancomycin stopped within 1 d of the final culture or CIDT result^d^	7 (1.7)	Supported
Vancomycin continued	5 (1.2)	Unsupported
Pathogen not susceptible or likely not susceptible to penicillin, ampicillin, or oxacillin	24 (6.0)	Supported
Pathogen identified from nonsterile site	112 (27.8)	NA
Signs or symptoms consistent with the reported infection site	106 (26.3)	NA
Pathogen susceptible or likely susceptible to penicillin, ampicillin, or oxacillin	36 (8.9)	NA
Severe or unspecified penicillin allergy	7 (1.7)	NA
Infection-specific treatment duration criterion met^c^	3 (0.7)	Supported
Infection-specific treatment duration criterion not met^c^	4 (1.0)	Unsupported
No severe or unspecified penicillin allergy	29 (7.2)	NA
Vancomycin stopped within 1 d of the final culture or CIDT result^d^	24 (6.0)	Supported
Vancomycin continued	5 (1.2)	Unsupported
Pathogen not susceptible or likely not susceptible to penicillin, ampicillin, or oxacillin	70 (17.4)	NA
Infection-specific treatment duration criterion met^c^	47 (11.7)	Supported
Infection-specific treatment duration criterion not met^c^	23 (5.7)	Unsupported
No signs or symptoms consistent with the reported infection site	6 (1.5)	Unsupported
**Total supported and unsupported vancomycin treatment**		
Supported vancomycin treatment	293 (72.7)	NA
Unsupported vancomycin treatment	110 (27.3)	NA

Abbreviations: CIDT, culture-independent diagnostic test; MRSA, methicillin-resistant *Staphylococcus aureus*; NA, not applicable.

^a^ Includes patients for whom cultures and CIDTs were not performed, patients for whom all culture and CIDT results were negative, and patients for whom culture and CIDT results were positive only for nonpathogens at the site of infection (eg, normal or mixed flora, yeast, or *Candida* species from a urine culture or respiratory tract culture).

^b^ Sepsis was defined using systemic inflammatory response syndrome criteria on the first day of vancomycin treatment based on 2 or more of the following: (1) temperature lower than 36 °C or higher than 38 °C, (2) heart rate greater than 90 beats per minute, (3) respiratory rate greater than 20 breaths per minute (or partial pressure of carbon dioxide, arterial <32 mm Hg), or (4) white blood cell count less than 4000 cells/mm^3^ or greater than 10 000 cells/mm^3^ or greater than 10% bands in addition to (1) systolic blood pressure less than 90 mm Hg, mean arterial pressure less than 65 mm Hg, or receipt of vasopressors or (2) lactate level greater than 2 mmol/L (to convert to milligrams per deciliter, divide by 0.111).

^c^ Treatment duration was defined as the duration of inpatient vancomycin treatment plus the anticipated duration of postdischarge vancomycin treatment. If postdischarge treatment duration was missing, it was estimated by determining the median days of postdischarge treatment among patients in the same step of the analysis pathway with the same duration of inpatient treatment. Data on other non-vancomycin antimicrobials were not collected. If not specified in the table, the following criteria were used: for lower respiratory tract infections and abdominal infections, fewer than 8 days was supported and 8 days or more was unsupported, and for skin and soft tissue infections, fewer than 11 days was supported and 11 days or more was unsupported. Treatment of any duration was considered supported for bloodstream infections; bone and joint infections; and ear, eye, nose, and throat infections.

^d^ The time from collection to final negative culture and CIDT results was estimated using the median time from collection to the final positive culture result.

^e^ Includes results of cultures or CIDTs performed 5 days before the initiation of vancomycin treatment through the last date of vancomycin treatment.

### Patients Included in Multiple AQUA Pathways

After exclusion of patients in the CAP or UTI pathway from the VANC and FQ pathways, 58 patients (3.7%) remained in multiple analysis pathways (VANC and FQ). Determinations in the 2 pathways were concordant for 32 of 58 patients (55.2%): 22 with supported and 10 with unsupported treatment. Discordant determinations (eg, unsupported for VANC and supported for FQ) were observed for 26 patients; after data for these patients were reviewed, 1 had an overall determination of supported treatment and 25, unsupported treatment. After discordant determinations were resolved, antimicrobial prescribing was determined to be supported for 690 of 1566 patients (44.1%; 95% CI, 41.6%-46.5%) and unsupported for 876 of 1566 patients (55.9%; 95% CI, 53.5%-58.4%) (eTable in the Supplement).

## Discussion

Among patients included in a multicenter hospital prevalence survey of health care–associated infections and antimicrobial use, a substantial percentage of CAP, UTI, FQ, and VANC treatment was unsupported by medical record data collected using a standardized approach (55.9% overall and as high as 79.5% for CAP). Common reasons for unsupported use included long duration, antimicrobial selection that deviated from guidelines, absence of documented signs or symptoms of infection, and lack of microbiologic evidence of infection.

Few recent, large studies have addressed inpatient antimicrobial prescribing quality.^21,22,23,24^ Comparison of our results with the results of these other studies is difficult because different approaches to data collection and different definitions of inappropriate or unnecessary prescribing of antimicrobials were used. In the other studies, antimicrobial prophylaxis and treatment were included and antimicrobial stewardship program personnel or other medical professionals collected the data and made determinations about antimicrobial prescribing quality.^21,22,23,24^ These studies also focused their assessments on antimicrobial prescriptions rather than infection syndromes. We focused solely on antimicrobials used to treat infections rather than including prophylaxis; did not require data collectors to have clinical or stewardship expertise; and used analysis pathways to categorize prescribing quality for 2 antimicrobial-based and 2 infection-based events.

Other studies have used terms such as *inappropriate* and *suboptimal* to describe prescribing quality but defined them in different ways. The use of multiple different definitions of appropriate and inappropriate prescribing is a particular challenge for hospital antimicrobial stewardship.^7^ Tribble et al^22^ considered suboptimal antimicrobial use to be inappropriate or appropriate with modification required; reasons included pathogen-drug mismatch, duplicate treatment (eg, 2 antimicrobials to cover anaerobes), unnecessary intravenous antimicrobial administration, overly broad coverage, and reasons classified as *other*. In contrast, the Australian Hospital National Antimicrobial Prescribing Survey defines inappropriate antimicrobial prescribing as being either suboptimal or inadequate.^24^ Suboptimal prescribing includes overly broad coverage, duplicate treatment, excessively long treatment, and failure to de-escalate on the basis of microbiology test results; inadequate prescribing includes antimicrobial use when antimicrobials are not needed and prescribing in which the antimicrobial selection, dose, route, or duration is deemed unlikely to treat the pathogen or likely pathogen.^24^ We opted to use the terms *supported* and *unsupported* as proxies for appropriate and inappropriate or unnecessary use because we did not require that data collection be performed by clinicians, and determinations were made through analysis pathways rather than by antimicrobial stewards using their clinical expertise and judgment to evaluate individual patient records.

We observed that the percentages of unsupported use were higher for infection-based events than for antimicrobial-based events. This finding may have been associated in part with our inclusion of more specific criteria in the infection-based analysis pathways according to treatment guidelines from professional societies, which tend to focus on types of infections. Although US infectious diseases and pharmacy professional societies have issued a guideline on therapeutic monitoring of VANC use for serious infections caused by methicillin-resistant *Staphylococcus aureus*,^25^ few national guidelines have focused on appropriate therapeutic uses of specific antimicrobials. In addition, it was not feasible to include specific criteria to cover aspects of prescribing for all possible infection types in the antimicrobial-based pathways. The larger percentages of supported FQ and VANC use compared with antimicrobial use for treatment of CAP and UTI may have been attributable to this exclusion. We believe that for the approach that we used, the infection-based assessments were more practical for implementation on a large scale and identified more opportunities for improving use.

One example of an opportunity for improvement suggested by our analysis is excessive treatment duration, which was the most common reason for unsupported CAP treatment and has been reported in multiple other studies.^26,27,28^ We calculated total treatment duration, including days of inpatient therapy plus the planned duration of postdischarge treatment. Current CAP guidelines recommend treatment for a minimum of 5 days, even if the patient has reached clinical stability before 5 days, stating that “as most patients will achieve clinical stability within the first 48 to 72 hours, a total duration of therapy of 5 days will be appropriate for most patients.”^17^ Exceptions are noted for CAP caused by methicillin-resistant *S aureus* or *Pseudomonas aeruginosa*, for which the recommended duration of treatment is 7 days.^17^ In our analysis, among 142 patients with CAP for whom duration of therapy was assessed, 103 (72.5%) were treated for at least 8 days. Among hospitalized veterans with uncomplicated pneumonia in 2013, 93.1% of patients with CAP received treatment for longer than the recommended duration.^26^ Among patients with CAP who were hospitalized in 2017 and 2018 in a Michigan Hospital Medicine Safety Consortium study, 71.3% received treatment for longer than the recommended duration.^27^ Given the harm associated with excessive treatment, studies are needed to establish effective approaches to reducing treatment duration, particularly after discharge.^27,28^

Absence of signs or symptoms of infection was another common reason for unsupported antimicrobial use among patients receiving UTI treatment. Recent updated guidelines^29^ have addressed the problem of inappropriate treatment of asymptomatic bacteriuria. Despite efforts to discourage treatment of asymptomatic bacteriuria, a large percentage of patients receiving UTI treatment in our analysis—approximately 38%—lacked documented signs or symptoms of infection. This is higher than the percentage observed in a similar analysis performed in 2011,^30^ in which approximately 23% of patients without a catheter who were being treated for UTI did not have documented signs or symptoms of infection. Results of a Veterans Health Administration study showed that among hospitalized patients with positive urine culture results in 2013 and 2014, 72% with asymptomatic bacteriuria received antibiotics.^31^ Interventions that incorporate elements such as education and clinical decision support have been shown to be associated with reductions in antimicrobial use for asymptomatic bacteriuria.^32,33,34^

### Limitations

This study has limitations. The numbers of hospitals and patients included in our analysis were limited and from just 10 states; consequently, the results may not be generalizable. We assessed antimicrobial treatment only and not surgical or medical prophylaxis; data on surgical prophylaxis from the Emerging Infections Program hospital prevalence survey have been published.^11^ Because of the complexity of evaluating inpatient antimicrobial use, we included only selected patients who were treated for a single infection type. Therefore, only 35.0% of patients receiving antimicrobial treatment during hospitalization were assessed, which is a limitation of an approach that does not use antimicrobial stewards to review and interpret data from individual patient records. Determining the appropriateness of antimicrobial use for the remaining 65% of patients, many of whom may have received antimicrobials for complicated infections, may be challenging with the use of our approach. In a small percentage of patients included in both the FQ and the VANC analysis pathways, discordant determinations had to be resolved by 1 of the authors (S.S.M.). Further refinement of the data collection and analysis pathways may reduce this need in future assessments. In addition, our assessment was based solely on medical record documentation. Incomplete documentation or failure to collect certain data, such as all antimicrobials received by patients during hospitalization in the FQ or VANC pathways, could have affected our results. We were not able to validate the results obtained using the analysis pathways with reviews of a subset of patient records by infectious diseases specialists or pharmacists. In addition, we did not assess risk factors for unsupported antimicrobial use.

## Conclusions

The findings suggest that standardized assessments of hospital antimicrobial prescribing quality can be used to estimate the appropriateness of antimicrobial use in large groups of hospitals. National assessments of prescribing quality to complement data on the volume of antimicrobial use in hospitals and improve prescribing practices may ultimately depend on the ability to access and analyze electronic health record data across hundreds or thousands of health care facilities. Until such approaches are feasible, the AQUA assessment may be repeated over time as part of intermittent prevalence surveys of health care–associated infections and antimicrobial use to describe changes in prescribing quality and estimate the effects of national antimicrobial stewardship initiatives.

## Notes

Supplement. **eMethods.** **eFigure 1.** Flow Diagram Depicting Patients Treated for Community-Acquired Pneumonia (CAP) Who Were Included in (N = 219) or Excluded From (N = 211) the Analysis **eFigure 2.** Flow Diagram Depicting Patients Treated for Present-on-Admission Urinary Tract Infection (UTI) Who Were Included in (N = 452) or Excluded From (N = 394) the Analysis **eFigure 3.** Flow Diagram Depicting Fluoroquinolone (FQ) Patients Included in (N = 550) and Excluded From (N = 518) the Analysis **eFigure 4.** Flow Diagram Depicting Patients Receiving Intravenous Vancomycin Treatment (VANC) Who Were Included in (N = 403) or Excluded From (N = 709) Analysis **eFigure 5.** Community-Acquired Pneumonia (CAP) Analysis Pathway **eFigure 6.** Present-on-Admission Urinary Tract Infection (UTI) Analysis Pathway **eFigure 7.** Fluoroquinolone Treatment (FQ) Analysis Pathway **eFigure 8.** Intravenous Vancomycin Treatment (VANC) Analysis Pathway **eTable.** Summary of Antimicrobial Prescribing Quality Across AQUA Events **eReferences.**
