# Errors in the Abstract Results; Results; Tables 1, 4, and 5; the Conflict of Interest Disclosures; and the Supplement

**DOI:** 10.1001/jamanetworkopen.2021.9526

**Published:** 2021-04-16

In the Original Investigation titled “Assessment of the Appropriateness of Antimicrobial Use in US Hospitals,”^1^ published March 18, 2021, there were multiple rounding errors in the Abstract Results, Results, and Table 1; an incorrect sentence order in the Results section; indentation errors in Tables 4 and 5; errors in the Conflict of Interest Disclosures; and data errors in the Supplement. This article has been corrected.^1^
