# Supplementary material for: Assessment of the Appropriateness of Antimicrobial Use in US Hospitals
Source: JAMA Netw Open. 2021 Mar 18;4(3):e212007. doi: 10.1001/jamanetworkopen.2021.2007 (PMC7974639; doi:10.1001/jamanetworkopen.2021.2007)
Supplement: Supplement. — eMethods. eFigure 1. Flow Diagram Depicting Patients Treated for Community-Acquired Pneumonia (CAP) Who Were Included in (N = 219) or Excluded From (N = 211) the Analysis eFigure 2. Flow Diagram Depicting Patients Treated for Present-on-Admission Urinary Tract Infection (UTI) Who Were Included in (N = 452) or Excluded From (N = 394) the Analysis eFigure 3. Flow Diagram Depicting Fluoroquinolone (FQ) Patients Included in (N = 550) and Excluded From (N = 518) the Analysis eFigure 4. Flow Diagram Depicting Patients Receiving Intravenous Vancomycin Treatment (VANC) Who Were Included in (N = 403) or Excluded From (N = 709) Analysis eFigure 5. Community-Acquired Pneumonia (CAP) Analysis Pathway eFigure 6. Present-on-Admission Urinary Tract Infection (UTI) Analysis Pathway eFigure 7. Fluoroquinolone Treatment (FQ) Analysis Pathway eFigure 8. Intravenous Vancomycin Treatment (VANC) Analysis Pathway eTable. Summary of Antimicrobial Prescribing Quality Across AQUA Events eReferences. [file jamanetwopen-e212007-s001.pdf]

Supplementary Online Content

Magill SS, O'Leary E, Ray SM, et al; Emerging Infections Program Hospital Prevalence Survey Team. Assessment of the appropriateness of antimicrobial use in US hospitals. *JAMA Netw Open*. 2021;4(3):e212007. doi:10.1001/jamanetworkopen.2021.2007

eMethods.

eFigure 1. Flow Diagram Depicting Patients Treated for Community-Acquired Pneumonia (CAP) Who Were Included in (N = 219) or Excluded From (N = 211) the Analysis

eFigure 2. Flow Diagram Depicting Patients Treated for Present-on-Admission Urinary Tract Infection (UTI) Who Were Included in (N = 452) or Excluded From (N = 394) the Analysis

eFigure 3. Flow Diagram Depicting Fluoroquinolone (FQ) Patients Included in (N = 550) and Excluded From (N = 518) the Analysis

eFigure 4. Flow Diagram Depicting Patients Receiving Intravenous Vancomycin Treatment (VANC) Who Were Included in (N = 403) or Excluded From (N = 709) Analysis

eFigure 5. Community-Acquired Pneumonia (CAP) Analysis Pathway

eFigure 6. Present-on-Admission Urinary Tract Infection (UTI) Analysis Pathway

eFigure 7. Fluoroquinolone Treatment (FQ) Analysis Pathway

eFigure 8. Intravenous Vancomycin Treatment (VANC) Analysis Pathway

eTable. Summary of Antimicrobial Prescribing Quality Across AQUA Events

eReferences.

This supplementary material has been provided by the authors to give readers additional information about their work.

© 2021 Magill SS et al. *JAMA Network Open*.

eMethods.

Community-acquired pneumonia analysis pathway

We included patients treated for community-acquired pneumonia (CAP) who were ≥18 years old with radiographic evidence of pneumonia in the first 5 hospital days plus signs or symptoms of pneumonia in the first 2 hospital days, who received ≥3 calendar days of inpatient pneumonia treatment and did not have other infections reported. Patients with certain underlying conditions were excluded, as noted in the main body of the manuscript (eFigure 1).

Radiographic evidence of pneumonia was defined by one or more of the following on any chest imaging study obtained in the first 5 hospital days: bronchopneumonia or pneumonia, new or worsening infiltrates affecting one or more lobes, air space density or opacity, consolidation, or cavitation. Radiology report language indicating "cannot rule out pneumonia" was also included. Patients whose sole radiographic finding was pleural effusion were not considered to have radiographic evidence of pneumonia.

Qualifying signs or symptoms of pneumonia in the first 2 hospital days included: fever, cough, dyspnea, oxygen saturation <90%, increased secretions or sputum production, hemoptysis or chest pain. Mental status change in the first 2 hospital days also qualified if it was accompanied by: 1) white blood cell count >10,000 cells/mm^3 on the first day of CAP treatment; 2) systolic BP <90 mmHg, mean arterial pressure <65 mmHg, or vasopressor administration on the first day of CAP treatment; 3) lactate >2 mmol/L (>18 mg/dL) on the first day of CAP treatment; or 4) ≥2 of the following on the first day of CAP treatment: temperature <36°C; heart rate >90 beats per minute; respiratory rate >20 breaths per minute (or $PaCO_2$ <32 mmHg); white blood cell count <4,000 cells/mm^3 or >10% bands.

When evaluating available microbiological data to determine if pneumonia pathogens were isolated from respiratory or sterile site cultures, we included the following culture sources: sputum, bronchoalveolar lavage, endotracheal aspirate, upper respiratory, blood, and pleura or pleural space. We evaluated the list of pathogens isolated from these culture sources and excluded the following from further consideration: *Bacillus* spp., *Candida* spp., coagulase-negative staphylococci, *Micrococcus* spp., *Propionibacterium* spp., and yeast. Data on bacterial colony-forming units (CFU) per milliliter (ml) were collected when available for cultures of lower respiratory tract specimens but were not used in the analysis pathway. Although culture-independent diagnostic test data were collected and included in the CAP pathway, very few positive results were observed. Examples of culture-independent diagnostic tests that were included in the pathway were: multiplex respiratory pathogen testing by polymerase chain reaction, serological testing for *Mycoplasma pneumoniae*, *Legionella* urine antigen testing, and upper respiratory specimen testing for methicillin-resistant *Staphylococcus aureus* by polymerase chain reaction. Because antimicrobial susceptibility data were frequently not available, we also determined whether identified pathogens were likely to have been susceptible to the antimicrobial medications being given to the patient.

When determining whether patients had received ≥3 calendar days of inpatient pneumonia treatment, we counted all antimicrobial medications given for CAP, from the earliest to the latest dates of administration. Days without antimicrobial medication administration were counted as part of the treatment course if they did not exceed 3 consecutive days. In some cases, patients who were reported to have been given post-discharge CAP treatment had missing duration of post-discharge treatment. To estimate total treatment duration for these patients (inpatient plus post-discharge), we determined the median duration of post-discharge treatment among those with known duration and the same duration of inpatient treatment and added this median value to the inpatient treatment duration for patients with missing post-discharge treatment duration.

For patients without microbiology data, the following antimicrobial medications on inpatient day 3 of CAP treatment were deemed to be guideline-similar:[1] 1) a macrolide (azithromycin, clarithromycin, erythromycin), or doxycycline, or a respiratory fluoroquinolone (moxifloxacin, gemifloxacin, levofloxacin); and/or 2) one of the following selected beta-lactams (amoxicillin, amoxicillin-clavulanate, penicillin G, cefpodoxime, cefuroxime, cefprozil, cefdinir, cefditoren, ceftriaxone, cefotaxime, ceftaroline, ertapenem, aztreonam, ampicillin, ampicillin-sulbactam). Combinations that included ≥2 of a macrolide, doxycycline and/or fluoroquinolone on day 3, continued for >1 day, were considered suboptimal. For example, a combination of azithromycin and levofloxacin given on day 3 and day 4 was considered suboptimal. Combinations that included ≥2 guideline-similar beta-lactam medications on day 3, continued for >1 day, were considered suboptimal. For example, a combination of ceftriaxone and cefpodoxime given on day 3 and day 4 was considered suboptimal. Combinations of guideline-similar medications with other antimicrobial medications on day 3 were considered suboptimal. As an example, a combination of ceftriaxone and metronidazole given on day 3, with no accompanying microbiology data, was considered suboptimal. Treatment with clindamycin or other beta-lactams (cefixime, cefaclor, cefadroxil, cephalexin, dicloxacillin) was also considered suboptimal.

© 2021 Magill SS et al. *JAMA Network Open*.

Urinary tract infection analysis pathway

We included patients treated for urinary tract infection (UTI) present on admission to the survey hospital who were not reported to be pregnant, neutropenic, or to have had a solid organ or hematopoietic stem cell transplant, and who received ≥1 calendar day of inpatient antimicrobial medications for treatment of UTI only. Patients with other infections during their hospitalization were excluded (eFigure 2). Neutropenia was assessed both as an underlying condition and on the first day of UTI treatment.

Qualifying signs or symptoms of UTI in the first 2 hospital days included: fever, urgency, frequency, pain or burning with urination, costovertebral angle pain or tenderness, suprapubic pain or tenderness, or visible blood in urine. Mental status change in the first 2 hospital days also qualified if it was accompanied by: 1) white blood cell count >10,000 cells/mm^3 on the first day of UTI treatment; 2) systolic BP <90 mmHg, mean arterial pressure <65 mmHg, or vasopressor administration on the first day of UTI treatment; 3) lactate >2 mmol/L (>18 mg/dL) on the first day of UTI treatment; or 4) ≥2 of the following on the first day of UTI treatment: temperature <36°C; heart rate >90 beats per minute; respiratory rate >20 breaths per minute (or $PaCO_2$ <32 mmHg); white blood cell count <4,000 cells/mm^3 or >10% bands.

When evaluating available urine culture data, we included each patient's first positive urine culture and first positive blood culture, plus any positive urine or blood cultures collected within 1 day of the first culture(s). We included the following urine culture sources: clean catch urine, catheter urine, other urine, fluid from the kidney, or fluid from a nephrostomy tube. Eligible positive urine cultures were defined as those with ≤2 bacteria isolated.[2] The following organisms were considered colonizers and excluded: yeast, *Candida* species, and normal or mixed flora.[2] Data on bacterial colony forming units per ml were collected but were not used in the analysis pathway. Positive blood cultures were defined as those with ≥1 National Healthcare Safety Network (NHSN)-defined recognized pathogen isolated.[3] Blood cultures positive for common commensals, as defined in the NHSN bloodstream infection protocol, were excluded. Antimicrobial susceptibility data were frequently not available, so we determined whether identified pathogens were likely to have been susceptible to antimicrobial medications being given to the patient.

In some cases, patients who were reported to have been given post-discharge UTI treatment had missing duration of post-discharge treatment. To estimate total treatment duration for these patients (inpatient plus post-discharge), we determined the median duration of post-discharge treatment among those with known duration and the same duration of inpatient treatment and added this median value to the inpatient treatment duration for patients with missing post-discharge treatment duration.

Fluoroquinolone analysis pathway

We included patients who received fluoroquinolone treatment (FQ) for at least 1 calendar day for a single infection type (eFigure 4). Sepsis was defined using Systemic Inflammatory Response Syndrome[4] criteria on the first day of FQ treatment: 2 or more of 1) temperature <36°C or >38°C; 2) heart rate >90 beats per minute; 3) respiratory rate >20 breaths per minute (or $PaCO_2$ <32 mmHg); or 4) white blood cell count <4,000 cells/mm^3 or >10,000 cells/mm^3or >10% bands, plus 1) systolic BP <90 mmHg, mean arterial pressure <65 mmHg, or on vasopressors, or 2) lactate >2 mmol/L (>18 mg/dL).

We evaluated microbiology data from cultures or culture-independent tests collected during the period starting 5 days before the start date of FQ treatment and ending on the last date of FQ treatment. We sought to apply similar rules to determining the eligibility of culture data for inclusion in the pathway as we applied in the CAP and UTI pathways. In addition, we included blood cultures that were positive for NHSN common commensals if there were two or more separate, positive cultures.[3] For some positive cultures, and for all negative culture and culture-independent diagnostic tests, the date of final microbiological test result was not available to use in determining whether FQ treatment was stopped promptly in response to test results. In these instances, we applied a proxy time of 3 days from specimen collection to final result date using the median time from collection to final result date among patients for whom these data were available. Fluoroquinolone susceptibility data were frequently not available, so we determined whether identified pathogens were likely to have been susceptible to the FQ being given to the patient.

In some cases, patients who were reported to have been given post-discharge FQ treatment had missing duration of post-discharge treatment. To estimate total treatment duration for these patients (inpatient plus post-discharge), we determined the median duration of post-discharge treatment among those with known duration and the same duration of inpatient treatment and added this median value to the inpatient treatment duration for patients with missing post-discharge treatment duration.

© 2021 Magill SS et al. *JAMA Network Open*.

Vancomycin analysis pathway

We included patients who received intravenous vancomycin treatment (VANC) for >3 calendar days for a single infection type (eFigure 3). Sepsis was defined using Systemic Inflammatory Response Syndrome[4] criteria on the first day of VANC treatment: 2 or more of 1) temperature <36°C or >38°C; 2) heart rate >90 beats per minute; 3) respiratory rate >20 breaths per minute (or $PaCO_2$ <32 mmHg); or 4) white blood cell count <4,000 cells/mm^3 or >10,000 cells/mm^3 or >10% bands, plus 1) systolic BP <90 mmHg, mean arterial pressure <65 mmHg, or on vasopressors, or 2) lactate >2 mmol/L (>18 mg/dL).

We evaluated microbiology data from cultures or culture-independent tests collected during the period starting 5 days before the start date of VANC treatment and ending on the last date of VANC treatment. We sought to apply similar rules to determining the eligibility of culture data for inclusion in the pathway as we applied in the CAP and UTI pathways. In addition, we included blood cultures that were positive for NHSN common commensals if there were two or more separate, positive cultures.[3] For some positive cultures, and for all negative culture and culture-independent diagnostic tests, the date of final microbiological test result was not available to use in determining whether VANC treatment was stopped promptly in response to test results. In these instances, we applied a proxy time of 3 days from specimen collection to final result date using the median time from collection to final result date among patients for whom these data were available. Vancomycin, oxacillin, and penicillin or ampicillin susceptibility data were frequently not available, so we determined whether identified pathogens were likely to have been susceptible.

Patients were considered to have a severe penicillin allergy based on the following reported reactions: hives or urticaria, blisters, wheezing, throat tightness, trouble breathing, angioedema, face, tongue, oral or lip swelling, or anaphylaxis. Free text fields clearly falling into any of these categories, or indicating any of the following, were also considered severe reactions: Stevens-Johnson syndrome, toxic epidermal necrolysis, Drug Reaction with Eosinophilia and Systemic Symptoms, fainting, syncope, joint swelling, liver failure, seizure, convulsions, encephalopathy, pancytopenia, thrombocytopenia, nephritis or acute kidney injury, and neuropathy. Examples of reported allergic reactions that were not considered severe: any gastrointestinal tract-related symptoms, other skin rash (including reports of cracked or peeling skin), fever, yeast infection, itching, and hand numbness.

In some cases, patients who were reported to have been given post-discharge VANC treatment had missing duration of post-discharge treatment. To estimate total treatment duration for these patients (inpatient plus post-discharge), we determined the median duration of post-discharge treatment among those with known duration and the same duration of inpatient treatment and added this median value to the inpatient treatment duration for patients with missing post-discharge treatment duration.

© 2021 Magill SS et al. *JAMA Network Open*.

eFigure 1. Flow diagram depicting patients treated for community-acquired pneumonia (CAP) who were included in (N=219) or excluded from (N=211) the analysis.

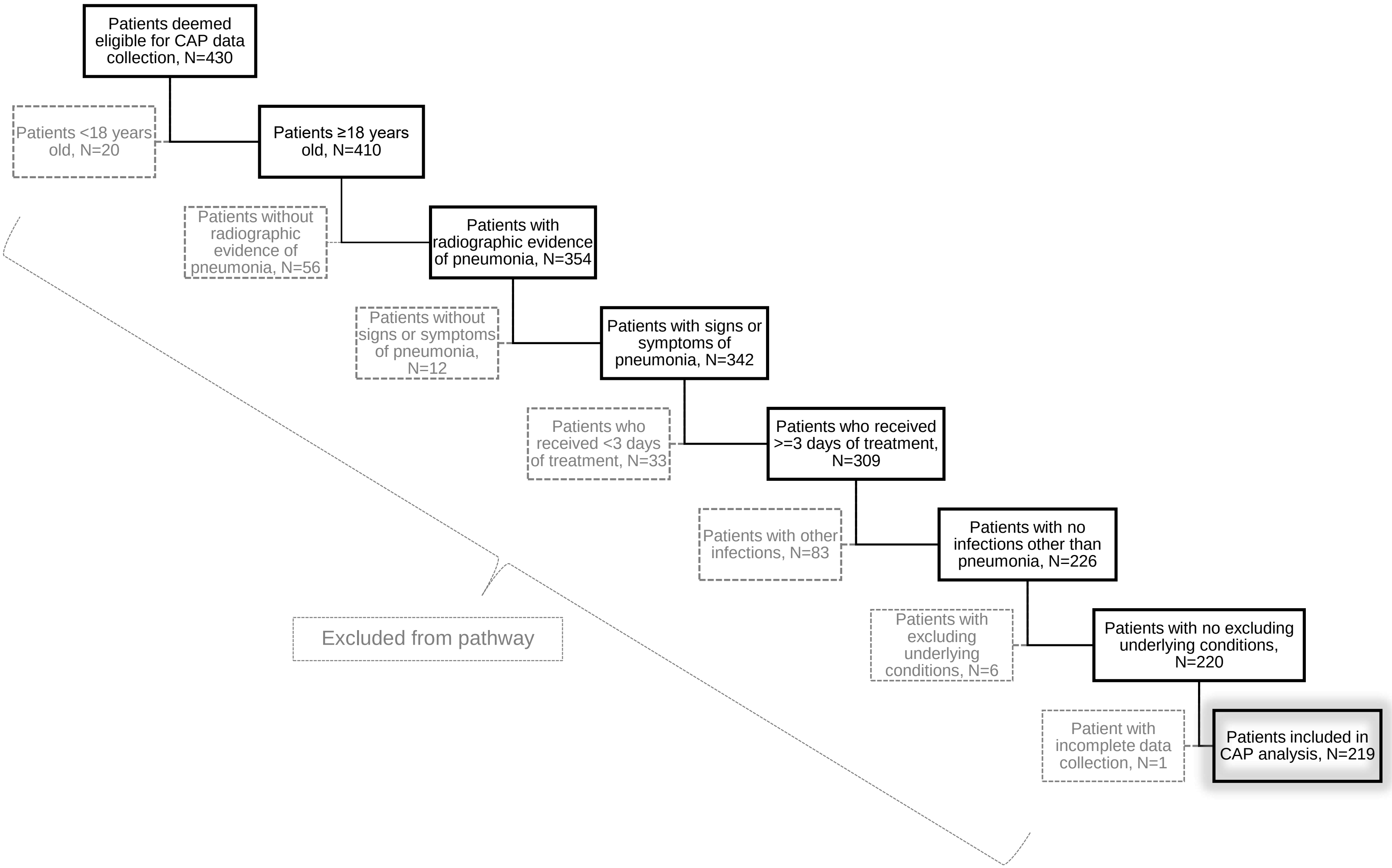

© 2021 Magill SS et al. *JAMA Network Open.*

eFigure 2. Flow diagram depicting patients treated for present-on-admission urinary tract infection (UTI) who were included in (N=452) or excluded from (N=394) the analysis.

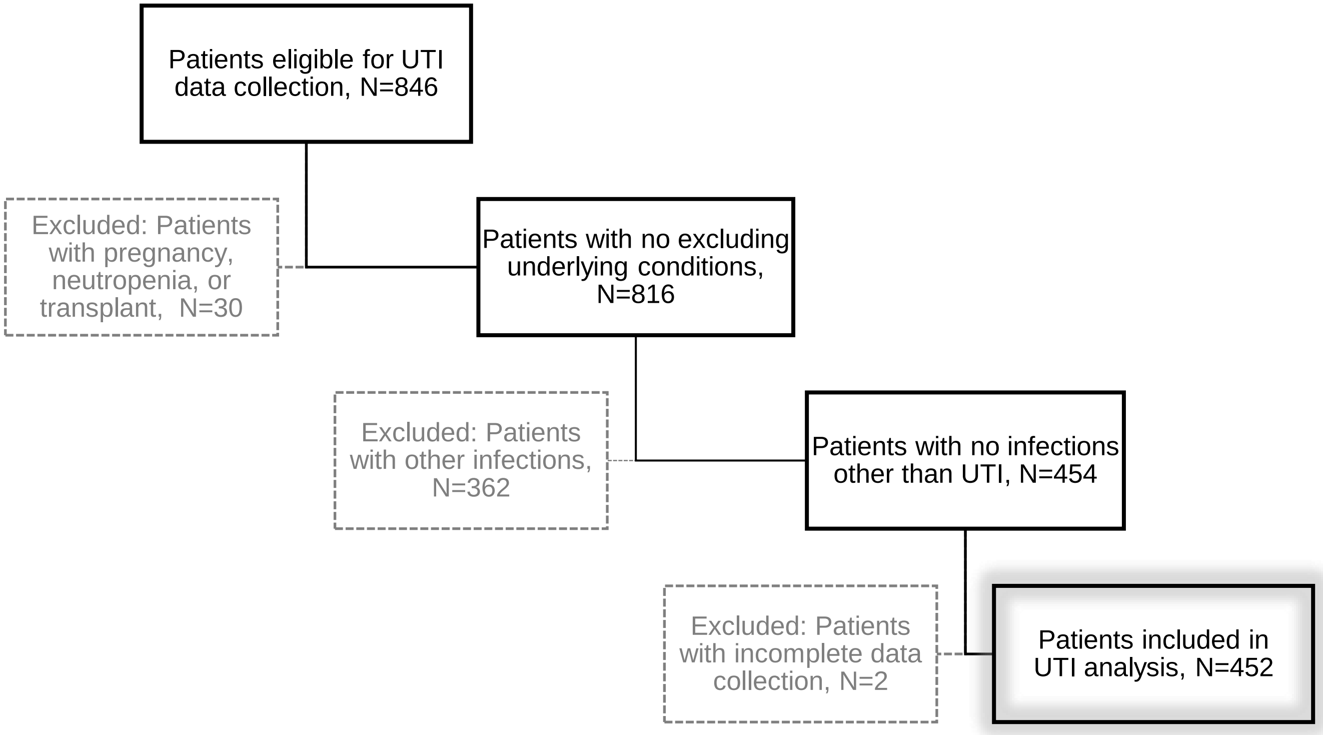

© 2021 Magill SS et al. *JAMA Network Open*.

eFigure 3. Flow diagram depicting fluoroquinolone (FQ) patients included in (N=550) and excluded from (N=518) the analysis.

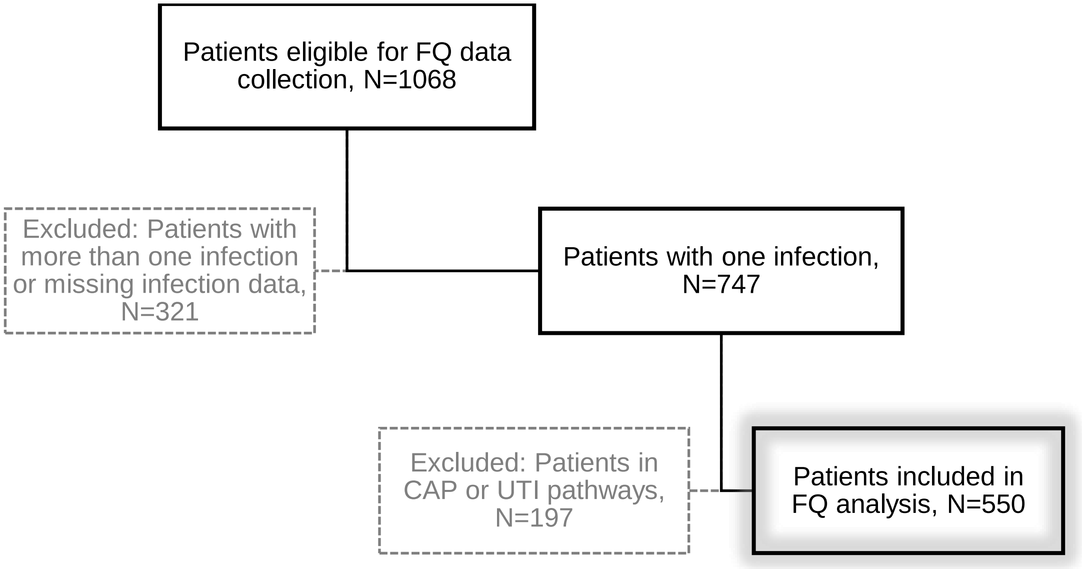

© 2021 Magill SS et al. *JAMA Network Open.*

eFigure 4. Flow diagram depicting patients receiving intravenous vancomycin treatment (VANC) who were included in (N=403) or excluded from (N=709) analysis.

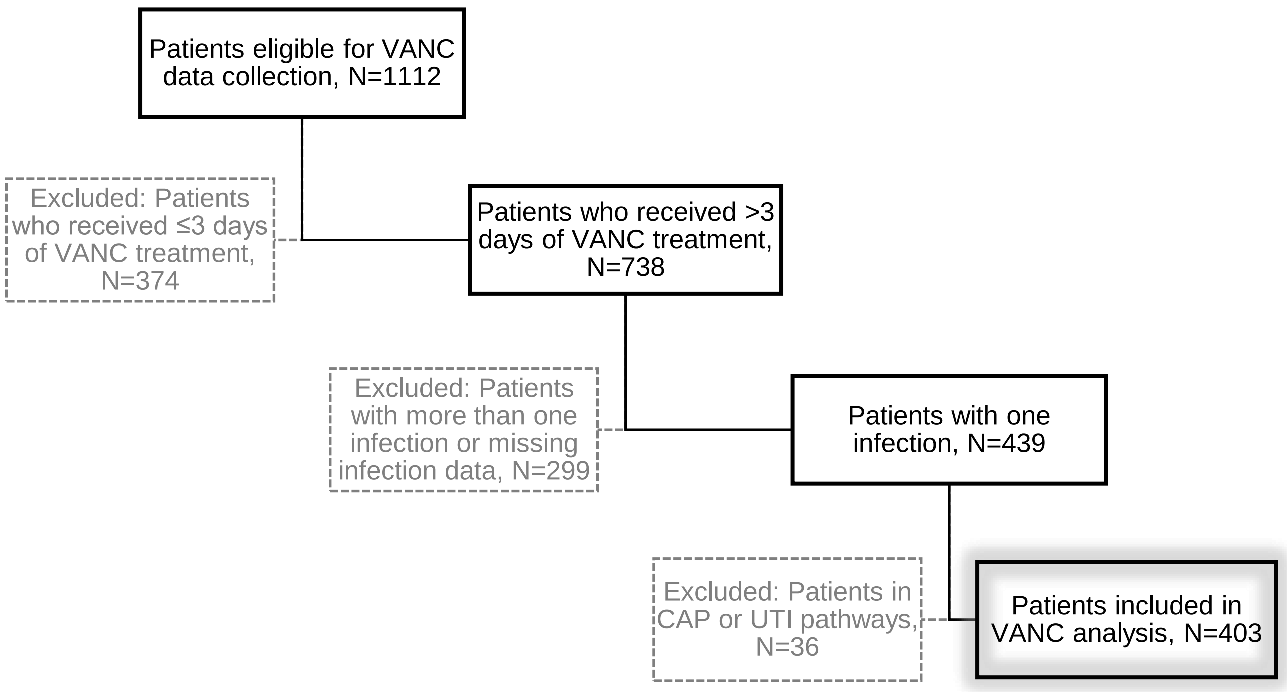

© 2021 Magill SS et al. *JAMA Network Open*.

eFigure 5. Community-acquired pneumonia (CAP) analysis pathway.

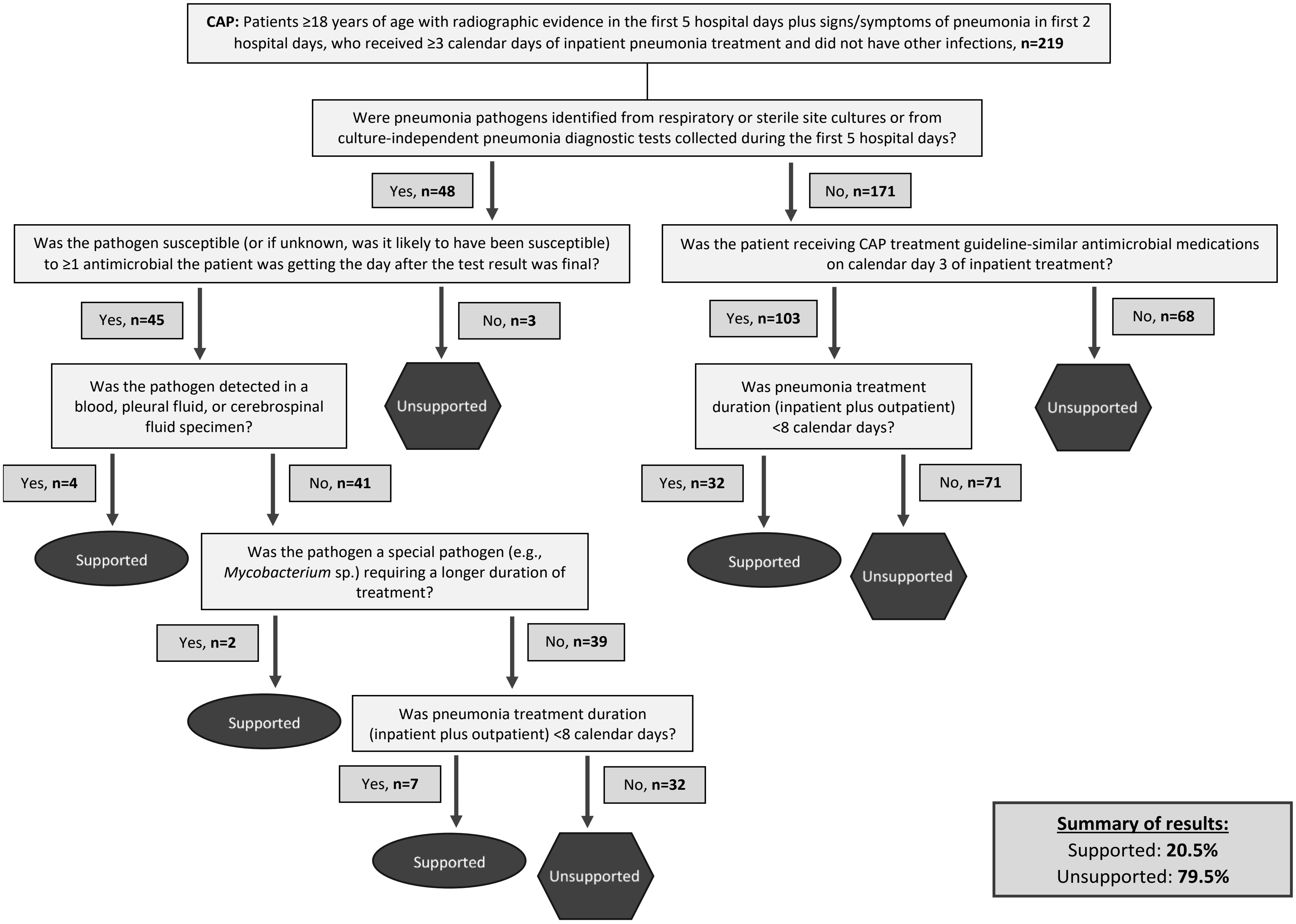

© 2021 Magill SS et al. *JAMA Network Open*.

eFigure 6. Present-on-admission urinary tract infection (UTI) analysis pathway.

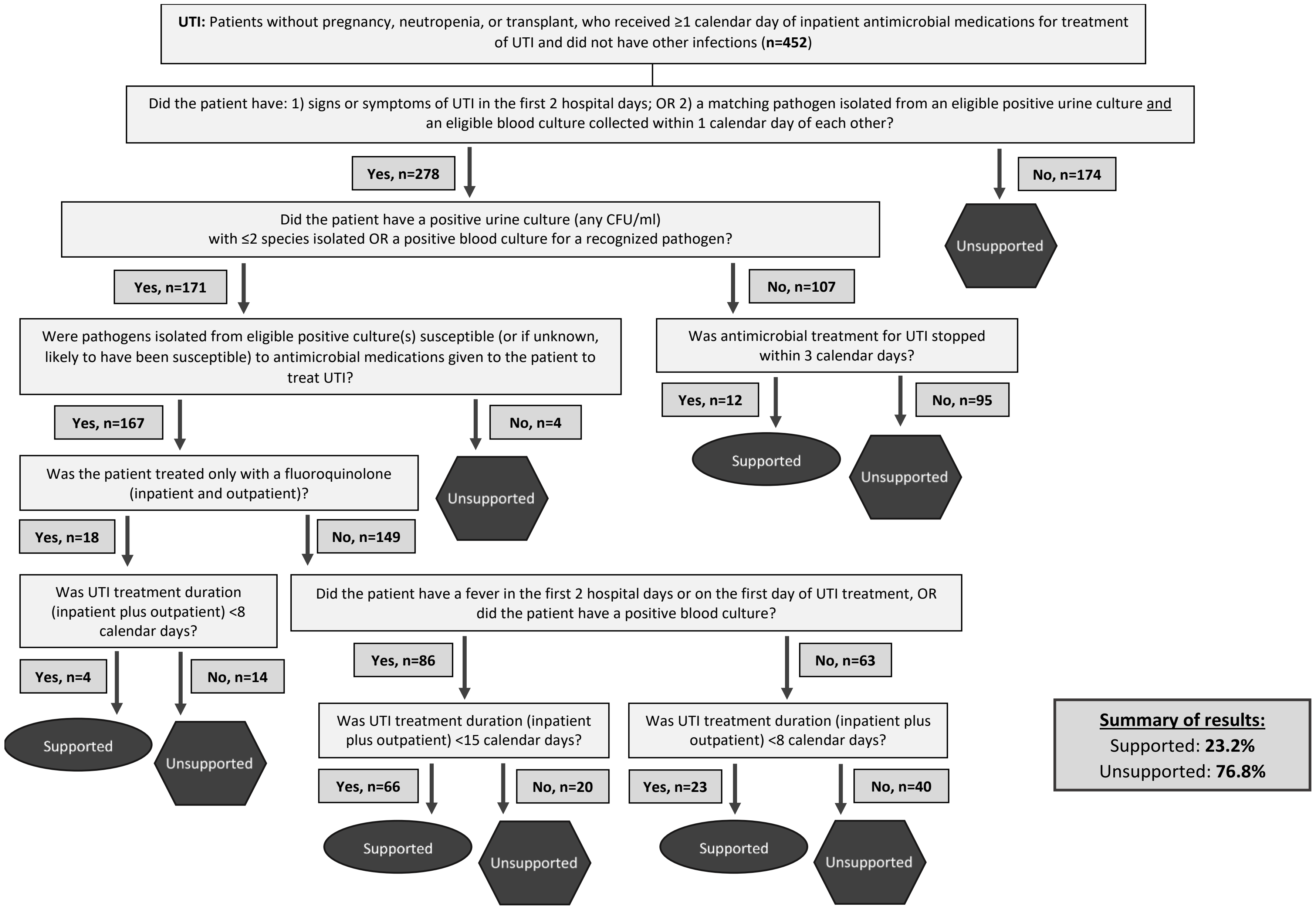

© 2021 Magill SS et al. *JAMA Network Open*.

eFigure 7. Fluoroquinolone treatment (FQ) analysis pathway.

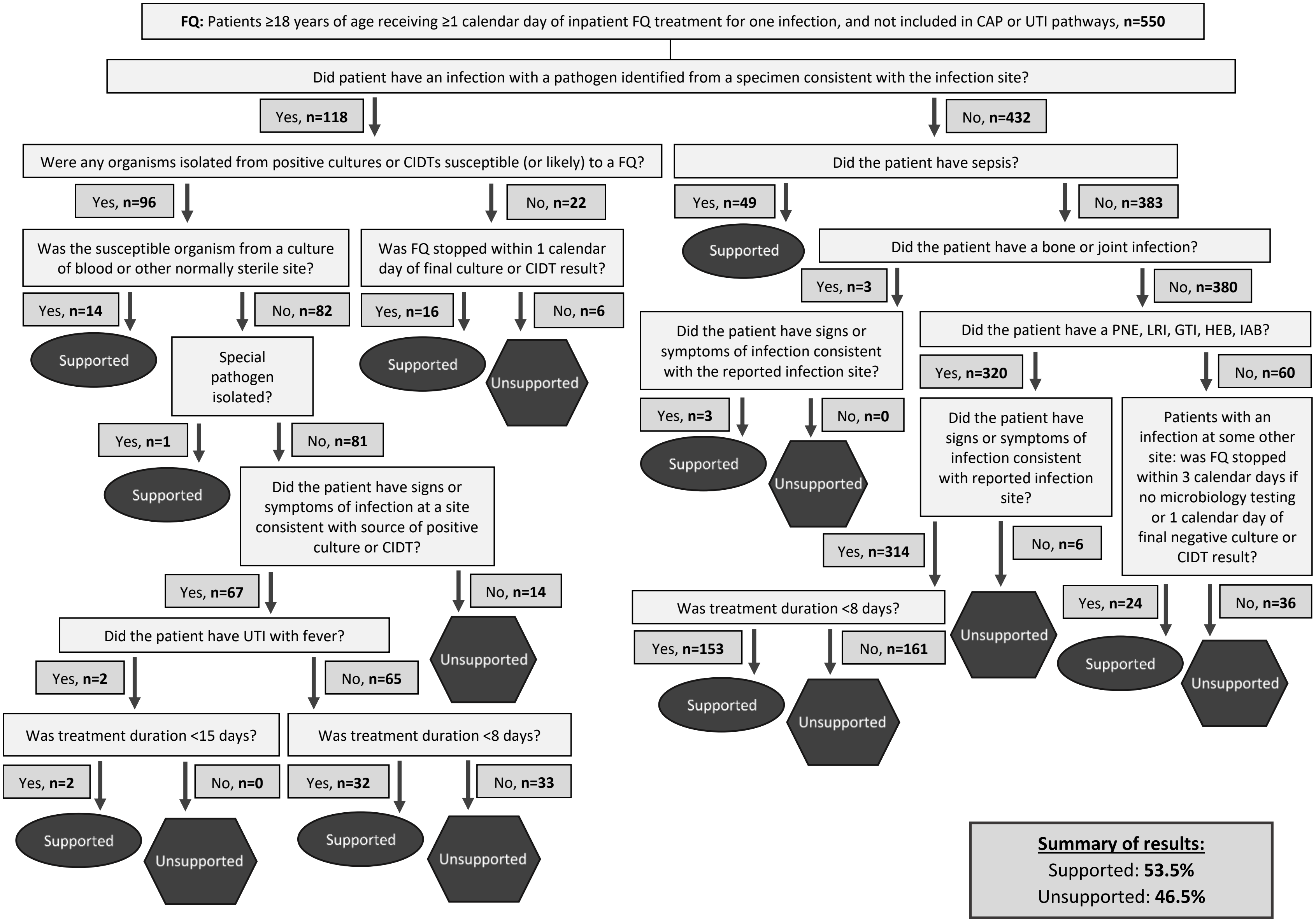

CIDT=culture-independent diagnostic test. PNEU=pneumonia. LRI=lower respiratory infection. GTI=gastrointestinal infection. HEB=hepatobiliary infection. IAB=intraabdominal infection.

© 2021 Magill SS et al. *JAMA Network Open*.

eFigure 8. Intravenous vancomycin treatment (VANC) analysis pathway.

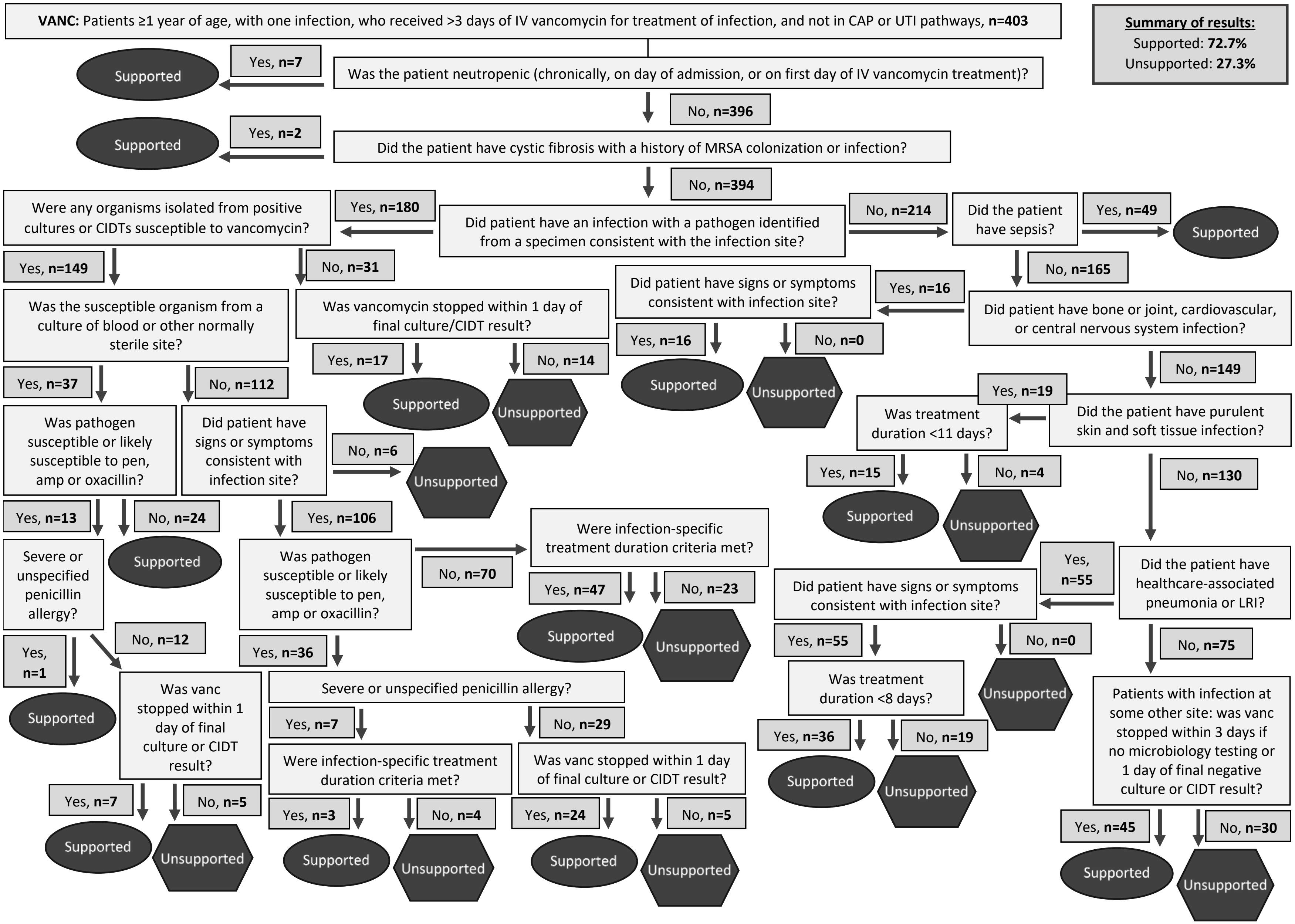

CIDT=culture-independent diagnostic test. MRSA=methicillin-resistant *Staphylococcus aureus*.
Pen=penicillin. Amp=ampicillin.

© 2021 Magill SS et al. *JAMA Network Open*.

eTable. Summary of antimicrobial prescribing quality across AQUA events.

		No. patients (%)	
**AQUA event(s)**	**Total**	**Prescribing was supported**	**Prescribing was unsupported**
CAP only	219	45 (20.5)	174 (79.5)
UTI only	452	105 (23.2)	347 (76.8)
FQ only	492	267 (54.3)	225 (45.7)
VANC only	345	250 (72.5)	95 (27.5)
VANC and FQ[a]	58	25 (43.1)	33 (56.9)
Total	1566	690 (44.1)	876 (55.9)

AQUA=Antimicrobial Quality Assessment. CAP=community-acquired pneumonia treatment. UTI=present-on-admission urinary tract infection treatment. VANC=intravenous vancomycin treatment. FQ=fluoroquinolone treatment.

[a]Among patients included in both FQ and VANC analysis pathways, discordant determinations were resolved to a single determination of supported or unsupported

© 2021 Magill SS et al. *JAMA Network Open*.

eReferences

1. Metlay JP, Waterer GW, Long AC, Anzueto A, Brozek J, Crothers K, et al. Diagnosis and treatment of adults with community-acquired pneumonia. An official clinical practice guideline of the American Thoracic Society and Infectious Diseases Society of America. *Am J Resp Crit Care Med*. 2019;200(7):e45-e67.
2. Centers for Disease Control and Prevention. National Healthcare Safety Network Surveillance for Urinary Tract Infections. Atlanta, GA: US Department of Health and Human Services, CDC; 2021. https://www.cdc.gov/nhsn/acute-care-hospital/cauti/index.html. Accessed January 21, 2021.
3. Centers for Disease Control and Prevention. National Healthcare Safety Network Surveillance for Bloodstream Infections. Atlanta, GA: US Department of Health and Human Services, CDC; 2021. https://www.cdc.gov/nhsn/acute-care-hospital/clabsi/index.html. Accessed January 21, 2021.
4. Bone RC, Balk RA, Cerra FB, et al. The ACCP/ SCCM Consensus Conference Committee. American College of Chest Physicians/Society of Critical Care Medicine. Definitions for sepsis and organ failure and guidelines for the use of innovative therapies in sepsis. *Chest*. 1992;101(6):1644-55.

© 2021 Magill SS et al. *JAMA Network Open*.
